# Cave plan repository: A standardized dataset for morphometric and geological analyses in speleology

**DOI:** 10.1016/j.dib.2026.112821

**Published:** 2026-06-10

**Authors:** Lazaridis Georgios, Vrettou Michaela, Dora Despoina, Karampelas Alexandros, Veni George, Tokmakidis Konstantinos, Trimmis Konstantinos, Vouvalidis Konstantinos

**Affiliations:** a Aristotle University of Thessaloniki, Thessaloniki, Greece; b National Cave and Karst Research Institute, USA

**Keywords:** Cave, Cave morphology, Cave morphometry, Cave dataset, Cave plan map

## Abstract

The Cave Plan Repository is a curated dataset of 562 standardized cave ground plans, designed to facilitate morphometric and geological analyses in speleology. The dataset integrates key metadata, including lithology and speleogenetic classification, enhancing its value for comparative studies in geomorphology, GIS-based cave modeling, and environmental science. By harmonizing cave maps from diverse sources into a uniform format, this dataset resolves issues of graphical inconsistency and accessibility, providing an open-access resource for interdisciplinary research. The repository enables systematic studies of cave morphology, contributing to the understanding of cave formation processes and supporting data-driven cave management practices. The dataset is publicly available via Mendeley Data [DOI: 10.17632/pt7skdpsfv.1].

## 1 Introduction

Cave morphometrics is the study and measurement of cave features, focusing on their shapes, sizes, orientations, and relationships within cave systems or even compare the total pattern of cave system development. This field plays a crucial role in understanding the geological processes that shape cave environments, and the dynamics of cave systems over time. Caves, as unique and often fragile environments, require precise and accurate data collection methods to assess their morphologies, which can differ significantly depending on geological history, water flow, and the biological activities within them.

Recent advancements in cave mapping technologies, including 3D scanning and laser-based techniques, have paved the way for highly detailed datasets that can characterize cave features at unprecedented levels of accuracy. However, the vast diversity of cave forms, especially in complex or remote systems are mapped with traditional spelological techniques following various methods and providing graphically heterogeneous designs. These factors necessitate the creation of comprehensive and standardized datasets that can be widely used for further research and applied science. A dataset of cave morphometrics can provide insights into a variety of scientific questions, ranging from paleoclimatic reconstructions to biodiversity conservation and even aiding in cave management practices. Furthermore, it developes a reference point for different studies that can use the same dataset.

The need for such datasets is especially critical as they can help resolve a number of ongoing challenges within cave science. Most importantly, they allow for a more uniform approach to cave classification, where researchers across different regions can compare cave structures and their respective features in a standardized way.

A recent review by Dora et al. [1] underscores the utility of morphometric datasets for addressing both fundamental and applied research challenges on speleology. This work highlights how systematic datasets can help mitigate the lack of comprehensive cave databases, which are often fragmented or inaccessible due to technical, logistical, and financial constraints. The dataset discussed in this work aims to fill this gap by providing a robust, open-access resource that can support interdisciplinary studies in speleology, geology, and environmental science.

## 2 Dataset Specifications

**Fig. 1:**
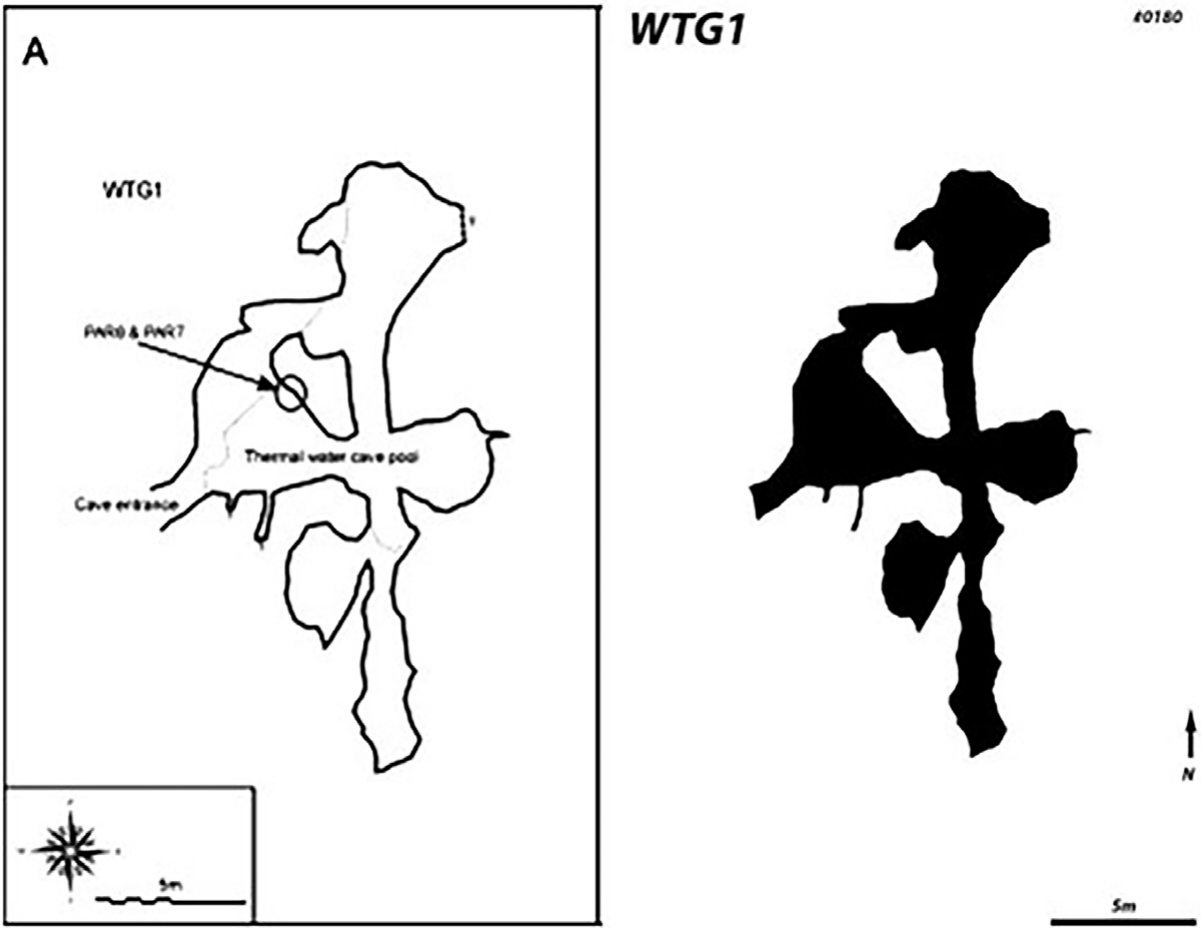
Example of cave map modification to fit the cave plan repository standards. Map of the WTG1 sulfuric acid cave (modified from [2]).

This dataset comprises standardized cave ground plans compiled from existing literature, enriched with key metadata to facilitate morphometric and geological analyses. Each cave plan has been transformed into a uniform **black-and-white format** with a **scale, north symbol, cave name, and key identifier**, ensuring comparability across different sources (Fig. 1). Additionally, all maps are presented in **A4 size**, optimizing them for digital and print applications. A significant enhancement of this dataset is the tagging of each cave according to its **lithology and speleogenetic classification**, providing critical insights into the geological context of cave development. This level of standardization and metadata integration enhances usability in geospatial, morphometrical and morphological research (Table 1).

**Table 1:** Specifications of the open access cave plan repository.

Specification	Description
Subject Area	Geomorphology / Speleology / GIS
More Specific Subject Area	Cave morphometrics
Type of Data	black-and-white format with a scale, north symbol, cave name, and key identifier
How Data Were Acquired	*Re*-designed from multiple sources
Data Format	jpeg files
Data Accessibility	Mendeley Data (see below for DOI)

## 3 Value of the Data

The value of this dataset lies in its accessibility, standardization, and research potential. Cave plans are often published in diverse formats, scales, and resolutions, making comparative analysis difficult. By harmonizing these maps into a consistent graphical and data framework, we enable researchers to perform quantitative morphometric studies, GIS-based cave modeling, and statistical correlations between cave morphology and geological setting. The added tags such as lithology and speleogenesis information further enhances its relevance for geomorphologists and speleologists seeking to understand cave evolution across different settings. This dataset is the first open-access dataset for cave morphometrics that serves as a valuable foundation for future studies in cave science, offering a centralized resource that can be integrated into various analytical workflows.

## 4 Data Description

The **cave plan repository** comprises a curated collection of 564 standardized cave ground plans, with each entry provided as a high-resolution image file. Each cave plan is assigned a unique numeric identifier (e.g., #0002), which also serves as the file name to ensure consistent indexing and easy retrieval. The accompanying metadata table includes the identifier, cave name, speleogenetic classification, bibliographic source, country or region, and optional notes, allowing users to filter and group entries according to geological or geographical criteria. When the primary source did not explicitly report the speleogenetic type, the field was left blank to avoid introducing interpretative bias. The dataset is specifically organized to support comparative morphometric studies, maintaining the original spatial orientation of each cave within a standardized **A4** layout. All data are hosted and publicly accessible via the **Mendeley Data** repository to ensure long-term availability and reusability for the scientific community.

## 5 Experimental Design, Materials, and Methods

This dataset provides a standardized and uniform collection of cave ground plans, addressing the common issue of inconsistent graphical representation in the literature. The development of the repository involved a systematic workflow focused on the collection, digitization, and harmonization of heterogeneous speleological data. The database includes 564 caves curated from an extensive review of global speleological literature and geological reports. The primary data were collected and modified from the works of Auler (3, b); Auler & Souza [4]; Audra (2017); Audra et al. [5]; Banzato et al. [6]; Bella & Gaál [7]; Bella et al. [8]; Beron et al. [9]; Blackwood [10]; Baciu et al. [11]; Banzato et al. [6]; Bella & Gaál [7]; Beron et al. [9]; Chervyatsova et al. [12]; Ciubotărescu & Onac [13]; Constantin [14]; De Vivo et al. [15]; De Waele et al. [16]; Doctor & Orndorff [17]; Dublyansky et al. [18]; D’Angeli et al. [[19], July]; D’Angeli et al. [20]; DuChene et al. [21]; Filippi et al. [[22], July]; Farrant & Harrison [23]; Forti [24]; Gázquez et al. [25]; Gilli [26]; Ginés et al. [27]; González-Ramón et al. [28]; Goran & Povară [29]; Gulley & Polk (2017); Häuselmann [30]; Hose & Rosales-Lagarde [31]; Jones [32]; Jones et al. [33]; Karban [34]; Karimi Vardanjani et al. [35]; Kadebskaya & Maksimovich [36]; Kempe et al. [37]; Klimchouk et al. [38]; Leél-Őssy [39]; Lazaridis [40]; Lazaridis et al. [2]; Martini [41]; Maslyn et al. [42]; Mylroie & Mylroie [43]; Nuțu-Dragomir & Dragomir [44]; Onac & Drăgușin [45]; Onac & Tămaş [46]; Osborne [47]; Osborne et al. [[48], July]; Palmer et al. [49]; Papiu [50]; Papiu & Onac [51]; Perşoiu & Onac [52]; Ponta (2019); Ponta et al. [53]; Ponta et al. [54]; Povară & Lascu [55]; Povară et al. [56]; Plan et al. (2012); Psotka & Papáč [57]; Roth [58]; Ros et al. (2017); Sauro et al. [[59], July]; Schindel & Gary [60]; Shanov & Kostov [61]; Stafford [62]; Springer (2018); Temovski [63]; Temovski et al. [64]; Tomuș & Breban (2019); Tomuș et al. (2019); Veres et al. (2019); Webb [65]; White [66]; White & White [67]; White et al. [68]; White (Ed.) (2017); Ponta & Onac [69] (2018).

The selection represents a broad geographical distribution, covering 29 countries including Albania, Austria, Algeria, Australia, Argentina, Bulgaria, Venezuela, Brazil, France, Germany, Greece, the United Kingdom, Israel, Iran, Spain, Italy, Kyrgyzstan, Mexico, the Bahamas, South Africa, Hungary, Ukraine, Russia, Romania, Slovakia, Sri Lanka, Czechia, Turkey, and the Philippines, as well as regions of the United States such as Arizona, Colorado, West Virginia, South Dakota, Texas, Florida, and Wyoming, and the territory of Puerto Rico. To eliminate graphical inconsistencies arising from different mapping eras, a rigorous digitization protocol was applied, transforming all primary maps into a uniform black-and-white (binary) format. All cave plans were processed using a consistent workflow. The original published maps were imported into ImageJ software and converted into a binary format through standardized thresholding. No geometric modifications were applied, and the original north arrow and scale bar were preserved. Each plan was then placed into a uniform A4 layout and exported as a high‑resolution JPEG file. This homogenization process enhances the accessibility of the plans for GIS-based analyses, quantitative morphometric studies, and machine learning applications. Of the total 564 entries, 470 caves have a defined speleogenetic origin and were categorized into specific types, including epigene (229), hypogene (123), flank margin (63), sulfuric acid (45), collapse room (6), and gypsum karst (4). The speleogenetic classificationsignificantly increases the analytical value of the dataset, allowing researchers to explore geological patterns across different cave-forming processes.

### Limitations

Although the Cave Plan Repository provides a standardized framework for speleological research, certain limitations inherent to the nature of compiled datasets must be acknowledged:

**Data Heterogeneity:** The dataset is derived from diverse primary sources spanning several decades. Consequently, the precision of the original surveys varies depending on the mapping techniques available at the time of the primary study.

**Two-Dimensional Representation:** The repository focuses exclusively on ground plans (2D). While these are essential for horizontal morphometry, they do not capture the vertical complexity or volumetric data of cave systems.

**Incomplete Metadata in Primary Literature:** Metadata such as precise entrance elevation or detailed local hydrological status were not consistently reported in all original publications. To maintain the integrity of the dataset, only verified information from the source materials was included.

**Geospatial Sensitivity and Precision:** Specific geographic coordinates (latitude/longitude) are often withheld in speleological literature to protect fragile cave environments and maintain conservation standards. This dataset prioritizes regional and country-level identification to align with these conservation practices.

**Bibliographic Bias:** The current collection reflects the availability of published cave plans in international and regional literature, which may result in higher representation for certain geographic areas.

### Data availability statement

Cave plan repository is acesible to the following DOI: Lazaridis, Georgios (2025), “cave plan repository”, Mendeley Data, V1.

## Declaration of Competing Interest

None.

## Data Availability

Mendeley DataCave morphology database (Reference data)
